# Mitochondrial COI Sequence Variations within and among Geographic Samples of the Hemp Pest *Psylliodes attenuata* from China

**DOI:** 10.3390/insects11060370

**Published:** 2020-06-14

**Authors:** Litao Guo, Feng Gao, Yi Cheng, Chunsheng Gao, Jia Chen, Zhimin Li, Tuhong Wang, Jianping Xu

**Affiliations:** 1 Institute of Bast Fiber Crops, Chinese Academy of Agricultural Sciences, Changsha 410205, China; guolitao@caas.cn (L.G.); redarkAN@163.com (F.G.); chengyi@caas.cn (Y.C.); gaochunsheng@caas.cn (C.G.); chenjia01@caas.cn (J.C.); lizhimin@caas.cn (Z.L.); wangtuhong@caas.cn (T.W.); 2 Department of Biology, McMaster University, Hamilton, ON L8S 4K1, Canada

**Keywords:** *Psylliodes attenuata*, hemp, genetic diversity, haplotype network, mitochondrial marker

## Abstract

The hemp flea beetle *Psylliodes attenuata* (Coleoptera: Chrysomelidae: Psylliodes) is a common pest of *Cannabis sativa*, including cultivars of both industrial hemp and medicinal marijuana. Both the larval and adult stages of this beetle can cause significant damages to *C. sativa*, resulting in substantial crop losses. At present, little is known about the populations of this pest, including its genetic diversity. In this study, we obtained 281 *P. attenuata* samples from nine field sites representing broad industrial hemp productions in China and analyzed their DNA sequences at the mitochondrial COI gene, the insect DNA barcode. Our analyses revealed a total of 48 haplotypes, with 28 being found only in one specimen each while the remaining 20 were shared by two or more specimens each. Of the 20 shared haplotypes, eight were shared among local populations often from far away locations, consistent with recent long-distance dispersals. However, the observed putative long-distance dispersals have not obscured the significant genetic differentiations among the regional populations from northeastern, eastern, central and southwestern China. Interestingly, haplotype network analyses suggest evidence for potential mitochondrial recombination in natural populations of this species. We briefly discuss the implications of our results on its evolution, center of diversity, route of spread, and pest management strategies in hemp fields.

## 1. Introduction

Industrial hemp (*Cannabis sativa* L.) is an ancient crop of Asian origin that has been traditionally cultivated in many regions of Europe and Asia, mainly as a source of textile fibers [1]. At present, aside from being used as textile fiber, *C. sativa* plants have also been developed into many other uses, including paper, biocomposites, functional foods, construction materials, cosmetics, biofuel and personal care [2,3]. For example, industrial hemp was found to be a valuable raw material for biofuel production [3]. Hemp seeds are a popular natural food, containing all the essential amino acids and necessary fatty acids required by humans [3,4]. Furthermore, *C. sativa* is of significant medical and recreational interests due to its productions of the psychoactive compound D-9-tetrahydrocannabinol (THC) and/or other cannabinoids such as cannabidiol (CBD). CBD and THC have shown pharmacological properties in the treatment of mood disorders, pain, cancer, diabetes, and inflammatory and neurodegenerative diseases [5,6]. As a result, there have been increasing interests to grow these crops. At present, different countries and different regions within certain countries have imposed restrictions regarding the varieties and cultivars of *C. sativa* that can be grown. For example, according to EU legislation (No 809/2014 of 17 July 2014), production of hemp is permitted if the content of THC is less than 0.2% [4,7]. In China, the growth of *C. sativa* is only allowed for the purpose of producing hemp fiber in jurisdictions with valid license and appropriate security measures.

Like all agricultural crops, hemp is susceptible to infections and damages by a variety of pathogens and pests. In *Cannabis* production areas, the hemp flea beetle *Psylliodes attenuata* is among the most common pests that can cause large crop losses [8]. *Psylliodes attenuata* (Koch) 1803 (Coleoptera: Chrysomelidae: Psylliodes), commonly known as hemp flea beetle or hop flea beetle, is geographically broadly distributed. It has been found throughout Eurasia, from the United Kingdom and France to the Middle East, eastern Siberia, Japan and China [8]. These flea beetles can damage hemp crops in both its larvae and adult stages, with adults causing more damages than grubs. The hemp flea beetles are monovoltine, reproducing only one generation per year. Overwintered adult beetles usually re-emerge in March–April. Before a hemp sprout appears, they mainly feed on hemp seeds. At low temperature, the adults concentrate on the soil surface and damage stems. Mating begins after a short period of additional feeding, with females laying eggs in the soil around host plants. Depending on the environmental condition, the eggs undergo embryogenesis, reaching 3 instars in 3–6 weeks, followed by pupation that occurs inside the soil which can last 1–7 weeks. During the larval stage, the beetles mainly feed on hemp roots. Young adult beetles usually emerge in August. At that time, they start consuming apical leaves, flowers, and immature seeds of hemp. Many flea beetles are polyphagous and feed on a wide range of plants. While the hemp flea beetle (*P. attenuata*) has been found on several plants such as hops and nettles, especially in the spring [9,10], it mainly feeds on hemps. Aside from being found in agricultural hemp fields, the hemp flea beetle is also listed as a major pest in indoor *Cannabis* growth facilities [11,12,13]. Indeed, due to its obligatory feeding on hemp, the hemp flea beetle has been suggested as an excellent candidate for eradicating illegal marijuana growth [14].

Despite its broad distribution and significant economic impact on the hemp industry, we know very little about the genetic diversity and phylogeography of hemp flea beetle. Molecular markers, such as those based on nuclear and organelle DNA sequences, have become increasingly popular for investigating the diversity and genetic relationships among populations of insects [15]. Specifically, the animal DNA barcode locus, a fragment of the mitochondrial cytochrome oxidase I gene (mt-COI), is often used as a molecular marker to determine the intraspecific and interspecific genetic relationship in insects [16]. As noted previously, there are several (potential) drawbacks in using the sequences of a single mitochondrial DNA fragment for genetic diversity studies, including (i) retention and incomplete lineage sorting of ancestral polymorphisms that could complicate phylogeographic patterns; (ii) inability to infer male-driven gene flow; (iii) highly susceptible to selective sweeps due to fitness advantage of a single nucleotide mutation (because the whole mtDNA genome is inherited together); (iv) inability to detect introgression following hybridization; and (v) potential paralogy due to transfer of mtDNA gene copies to the nucleus, causing difficulties in obtaining clean sequences and in data analyses [17]. Nonetheless, there are also advantages in selecting mitochondrial DNA in population and evolutionary investigations, including its multiple copy nature per cell and a fast rate of evolution in animals. These two features make the mitochondrial genes easier to amplify through PCR than nuclear genes (especially when dealing with small quantities of materials) and with a higher level of polymorphisms and greater discriminatory power than nuclear genes of similar lengths [16]. Furthermore, the functional conservation of mt-COI and their lack of introns in animals ensure that primers can be designed to work with diverse range of species and that the obtained DNA sequences can be easily aligned for population genetic and phylogenetic studies [16]. This is especially important for non-model organisms that have been little investigated, such as the hemp flea beetle. Indeed, hundreds of papers have been published based on mitochondrial DNA sequences, including COI sequences, for biodiversity and phylogeographic studies. For example, at the above-species level, DNA sequence variations at COI locus have been used to assess systematics and taxonomy of insects in Pseudococcidae [18] and Lepidoptera [19]. Furthermore, COI sequences have been used to investigate the invasion history of the sirex woodwasp *Sirex noctilio* populations in China [15] and to identify the genotypes of three-cornered alfalfa hopper *Spissistilus festinus* in the United States [20].

In this study, we collected samples of *P. attenuata* populations from six provinces in China and obtained their DNA sequences at the mitochondrial COI gene. The sequence data were used to infer the diversity and relationships among geographic populations of *P. attenuata* in China. The relevance of our results to the control of this pest in hemp fields will be discussed.

## 2. Materials and Methods 

### 2.1. Sample Collection and Identification of *Psylliodes attenuata*

For this study, we broadly sampled hemp flea beetles in four hemp growth regions in China: northeastern China (including Heilongjiang and Jilin provinces), central-east China (Shandong and Anhui provinces), south-central China (Hunan province), and southwestern China (Yunnan province). Within four provinces Heilongjiang, Anhui, Hunan, and Yunnan, we sampled three sites from each province. For the remaining two provinces Shandong and Jilin, only one site was sampled in each. Among the 14 sampled sites, nine were found to be infested by *P. attenuata*. The remaining five, including two each in Hunan and Yunnan provinces and one in Heilongjiang province, did not have *P. attenuata* at the time of our sampling. The sampling site information, including hemp cultivar and the number of hemp flea beetles used for subsequent analyses, are presented in Table 1. At each site where the hemp flea beetles were present, we covered the hemp fields broadly in all four directions from the center of the site, with individual collections located at least two meters from each other. In addition, we also surveyed the surrounding fields of other crops and forests for hemp flea beetles, but we did not find any. The collected adult beetles were soaked in 99.7% anhydrous alcohol for storage. These beetles were identified as hemp flea beetles based on their morphological features [8].

### 2.2. DNA Sequencing

A total of 281 adult hemp flea beetles were analyzed. For each beetle, its genomic DNA was isolated using the E.Z.N.A.^®^ Insect DNA kits (Omega Bio-Tek) according to the manufacturer’s instructions. The extracted DNA samples were stored in an −20 °C freezer until use. The quality and quantity of each extracted DNA sample was assessed using Nanodrop (Thermo Scientific Nanodrop 2000). To obtain the COI barcoding fragment, the general invertebrate COI amplification primers (the forward primer LCO1490 (5′-GGTCAACAAATCATA AAGATATTGG-3′) and the reverse primer HC02198, (5′-TAAACTTCAGGGTGACCAAAAAAT CA-3′)) were used to amplify the target sequence of *P. attenuata* in PCR reactions [21]. The PCR reactions were performed in 25 μL total volume, including 1 μL of DNA template, 2.5 μL of 10 × PCR buffer, 4 μL of dNTP mixture (2.5 mM each), 0.2 μL of La-Taq (Takara), 0.75 μL of each primer (10 mM), and 15.8 μL of ddH_2_O. The thermocycler procedure was as follows: initial denaturation for 1 min at 94 °C, followed by 20 cycles of increasing the annealing temperature by 0.5 °C every cycle at 94 °C for 1 min, annealing at 45 °C for 90 s, and extension at 72 °C for 1 min, and 20 cycles of denaturation at 94 °C for 1 min, annealing at 55 °C for 90 s, and extension at 72 °C for 1 min, with a final extension at 72 °C for 5 min [16]. PCR products (5 μL) were analyzed by electrophoresis on a 1.5% (*w/v*) agarose gel (1 × TAE), alongside a DNA marker (Marker III, TIANGEN, Beijing, China). After electrophoresis at 150 V for 40 min, the PCR products were visualized using ethidium bromide and ultraviolet light. Finally, products clearly visible after electrophoresis were sent to a commercial company (TSINGKE, Shanghai, China) for sequencing in both directions.

### 2.3. Data Analyses

All COI sequences were checked manually in BioEdit v7.0.9.0 to assess their quality [22]. The alignment was done using ClustalW implemented in MEGA v6.06 with default parameters, and no pseudogene nor stop codon were detected in the sequences [23,24]. 

Common parameters of genetic diversity, including the number of haplotypes, number of private haplotypes, and the haplotype and nucleotide diversities, were calculated using DNAsp v6 [25]. The haplotype network was built in PopART [26]. Phylogenetic trees were constructed using the neighbor-joining (NJ) method in MEGA v6.0 software [24]. The robustness of individual branches of the phylogeny was tested by the bootstrap method. Rates of change among lineages were presumed to be gamma distributed (G), and the bootstrap of each branch of the phylogenetic tree was subjected to *n* = 1000 replications.

We investigated the geographic distributions of mitochondrial COI single nucleotide polymorphisms and haplotypes within and among geographic populations, using the program GenAlEx 6.5 [27]. The contribution of geographic isolation to the total nucleotide polymorphisms in the *P. attenuata* samples was estimated based on the analysis of molecular variance (AMOVA). Genetic differences among pairs of geographic populations were assessed based on the traditional *F_ST_* values. In addition, the relationship between genetic distance and geographic distance was assessed using the non-parametric Mantel test, based on data on the two distance measures between all pairs of specimens (281 × 280/2 = 39,340 pairs of datapoints). In the Mantel test, the pairwise genetic distance between specimens was calculated as the number of nucleotide differences between their COI sequences, while the pairwise geographic distance was calculated as direct physical distance based on their longitudinal and latitudinal coordinates that was transformed logarithmically. The AMOVA, pairwise population *F_ST_*, and Mantel test were conducted using the GenAlEx V 6.5 software, with statistical significance for both the AMOVA and *F_ST_* values derived based on 1000 permutations [27]. 

## 3. Results

### 3.1. Identification and Characterization of Mitochondrial COI Sequences

In this study, a total of 281 *P. attenuata* specimens were analyzed for their mitochondrial COI sequence polymorphisms. These 281 specimens were captured in nine sites in six provinces. Our BLAST search comparisons for each of the 281 COI sequences revealed that our sequences shared >99% of nucleotide identity to that of a known COI DNA sequence from *P. attenuatus* (GenBank accession no. KM440790.1, syn. *P. attenuata*). Thus, the sequencing results confirmed that the morphological identifications were correct and that all our samples belonged to the same species. The obtained COI DNA sequences were aligned, with the total alignment contained 645 bp. Among the 645 aligned nucleotide sites for the 281 specimens, 600 were conserved, 45 were variable, and there was no insertion or deletion. Of the 45 variable sites, 16 were singleton sites and 29 were parsimony-informative, with the less frequent nucleotide shared by at least two specimens at each of the 29 sites. The average base compositions of A, G, C, T in the COI gene fragments were 31.0%, 15.40%, 17.40%, and 36.10%, respectively, with the average content of A + T about twice of that of C + G.

### 3.2. COI Haplotype Distributions

Based on nucleotide polymorphisms, the 281 COI sequences were grouped into 48 haplotypes. The detailed distributions of the 48 haplotypes are shown in Table 2. Among the 48 haplotypes, 28 were found only in one specimen each while the remaining 20 were shared by two or more samples each. The 28 singleton haplotypes were distributed across all nine local populations, i.e., each local population has its own singleton haplotypes. Of the 20 shared haplotypes, 12 were exclusively shared among specimens from within the same local population while the remaining eight (H1, H6, H7, H14, H19, H24, H33 and H39) were shared between and among local populations. Furthermore, all these eight haplotypes were shared between provinces, with six shared between different geographic regions (H1, H6, H7, H14, H19, and H24), while two haplotypes (H33 and H39) were shared between two provinces in northeastern China.

Among the 48 haplotypes, two, H1 and H33, were especially abundant, representing 91 and 61 specimens, respectively (Table 2). As mentioned above, haplotype H33 was shared among all three local populations in two provinces, Heilongjiang and Jilin, in northeastern China. Indeed, H33 was only found in northeastern China and account for over two-thirds (61/90) of all specimens from this region. In contrast, haplotype H1 was more broadly distributed, found in seven local populations in four provinces, including in northeastern (Heilongjiang province), central-east (Shandong and Anhui provinces), and central-south China (Hunan province). Among the seven local populations, three were dominated by the H1 haplotype, accounting for 25, 26, and 27 specimens, respectively (out of a total of 30 samples at each site). Significantly, all three sites dominated by the H1 haplotype were in Anhui province, and the three sites were located close to each other. 

As described above, eight of the 48 COI haplotypes were shared between at least two of the nine local populations of *P. attenuata*. These eight haplotypes accounted for >70% (198/281) of our total specimens (Table 2). The remaining 83 (281 – 198 = 83) specimens were distributed among the 40 COI haplotypes with each of the 40 haplotypes found only in one of the nine local populations. Interestingly, each of the nine local population has three or more private haplotypes (Table 3), with the AH1, AH2, AH3, and HRB local population having three private haplotypes each, DQ with four private haplotypes, HN with five private haplotypes, SD and YN with six private haplotypes each, and JL with seven private haplotypes. Of special note was that all 29 specimens from the YN site had COI haplotypes found only at this site and the six haplotypes at this site were not shared with any other geographic population (Table 2).

### 3.3. Nucleotide and Haplotype Diversities within Local Populations of Psylliodes attenuata

Aside from the differential distributions of COI haplotypes, the nine local populations also differed in the number and diversity of COI haplotypes (Table 3). Briefly, the number of COI haplotypes ranged from 4 to 11 per local population, with the fewest number (4) found in two local populations in Anhui province. The highest number (11) of COI haplotypes was found in the local population in Shandong province. Not surprisingly, the local population in Shandong also had the highest COI haplotype diversity, at 0.871. However, though the two local populations in Anhui with four haplotypes each had among the lowest haplotype diversities (at 0.193 and 0.251, respectively), there was no strict overall correlation between haplotype number and haplotype diversity among the nine local populations.

Similar to haplotype number and haplotype diversity patterns, there was a range of nucleotide diversities among the nine local populations. Nucleotide diversity in a population refers to the mean percentage nucleotide difference between pairs of sequences in the population. Here, the highest nucleotide diversity was found in the local population in Hunan province, followed by those in Shandong and Yunnan, with the lowest in Anhui province. Tajima’s D test results indicated that three local populations (Shandong, Hunan, and Yunnan) had positive D values, consistent with balancing selection in these three local populations. The remaining six local populations had negative D values, consistent with recent selective sweep, or population expansion after a recent bottleneck. However, only three of the nine D values were statistically significantly different from 0 (i.e., “0” means that the population was evolving in a mutation-drift equilibrium, with no evidence of selection). The three local populations included one each from Anhui, Jilin, and Heilongjiang (Daqin) (Table 3), with all three local populations showing evidence of recent selective sweep and/or population expansion after a recent bottleneck.

### 3.4. Phylogenetic Relationships among Haplotypes

**Figure 1:**
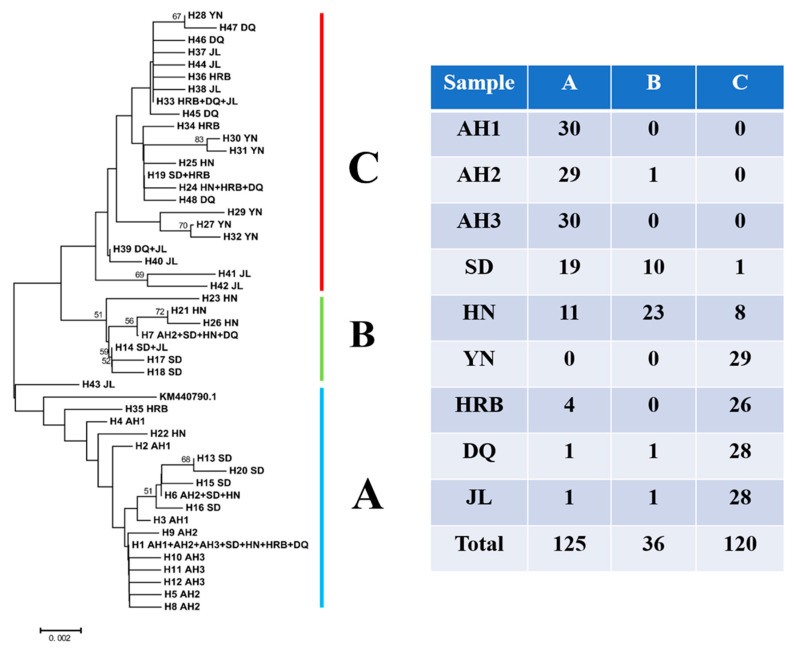
A Neighbor-Joining tree showing the phylogenetic relationships among the 48 haplotypes of mitochondrial *COI* gene of *Psylliodes attenuata* from China. The haplotype numbers (e.g., H1) correspond to those described in Table 1. The letters (and numbers in certain circumstances) following the haplotype number represent geographic distributions of each haplotype. A, B, and C are three large COI haplotype groups and their summary geographic distributions are listed in the table to the right.

**Figure 2:**
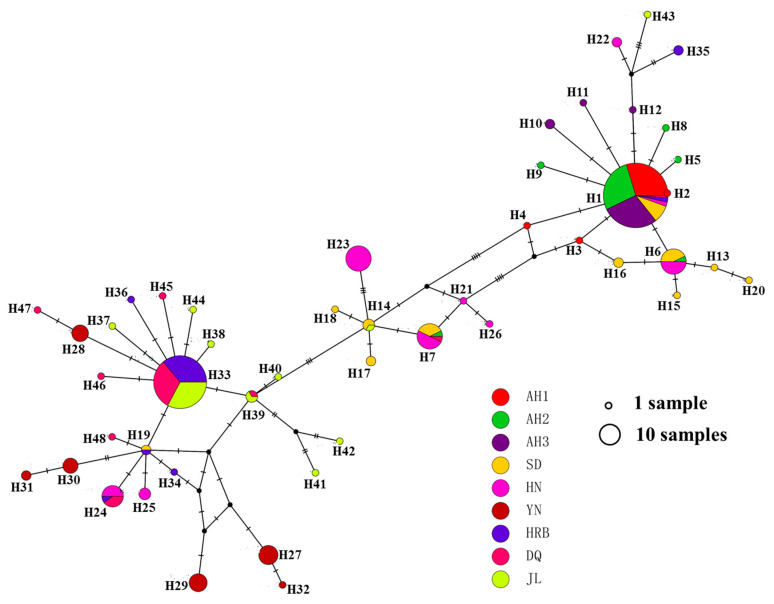
Haplotype network of mitochondrial COI sequences of 281 specimens of *Psylliodes attenuata* from nine local populations in China. Short tick lines between haplotypes show the number of mutations. The size of each circle is proportional to the number of specimens for each haplotype while the color(s) represents the geographic distributions of each haplotype.

The relationships among mitochondrial COI haplotypes are illustrated in Figure 1 and Figure 2. Figure 1 shows the Neighbor-Joining (NJ) tree of the 48 haplotypes while Figure 2 shows the haplotype network relationships among these haplotypes. The unrooted NJ tree showed that the 48 haplotypes were clustered into three large groups, A, B and C, representing 18, 7 and 23 haplotypes, respectively (Figure 1). All but one of the 90 specimens from Anhui province (AH1: 30, AH2: 29 and AH3: 30) clustered into group A. The vast majority of samples from Yunnan (YN: 29), Heilongjiang (HRB: 26, DQ: 28) and Jilin (JL: 28) provinces were clustered in group C. However, the samples from Shandong province (SD) were frequently distributed in groups A and B, while the samples from Hunan province (HN) were almost equally frequently distributed in groups A, B and C (Figure 1). In combination with the number of private haplotypes at each local population (the highest was seven in Jilin), the broad phylogenetic distributions of haplotypes from Jilin (in all three major groups) and a private haplotype H43 from Jilin being closest to the root of the mid-point rooted tree suggested that Jilin might be the centre of origin for hemp flea beetles in China.

The haplotype network analyses revealed a similar pattern but with additional information and complexity (Figure 2). Specifically, first, the two common haplotypes, H1 and H33, were linked with a diversity of less frequent haplotypes by one or a few mutations (e.g., H2 to H6 and H8 to H12 linked with H1; and H36-H38, H44-H46 linked with H33). Second, looped networks were observed for several groups of observed haplotypes and/or inferred intermediate haplotypes (Figure 2). For example, haplotypes H1, H3, H6, and H16 represent all four possible combinations of two alleles at two polymorphic nucleotide sites (Figure 2).

### 3.5. Population Relationships

Our haplotype distribution data described above indicated both unique and shared mitochondrial COI haplotypes within and among local and regional populations of *P. attenuata*. The results suggest potential genetic differentiation among the geographic populations as well as evidence for recent migrations and/or incomplete lineage sorting. To further quantify the contributions of geographic separation to the observed nucleotide polymorphisms, we estimated the pairwise population genetic relationships using the standard Wright’s *F_ST_* using the program DnaSP 6.0. Our results showed that geographic separation contributed significantly to the observed sequence variations, with an *F_ST_* of 0.59635 (*p* < 0.001). However, between individual pairs of local populations, the *F_ST_* values ranged widely, from −0.01205 to 0.88233 (Table 4). For example, among the three local populations in Anhui province, their pairwise *F_ST_* values were all very low and genetically indistinguishable. Similarly, among the three local populations in northeastern China, their *F_ST_* values were also low and genetically very similar to each other. However, all other pairwise comparisons yielded statistically highly significant genetic differentiations, including all comparisons involving the local populations from Shandong, Hunan and Yunnan (Table 4).

**Figure 3:**
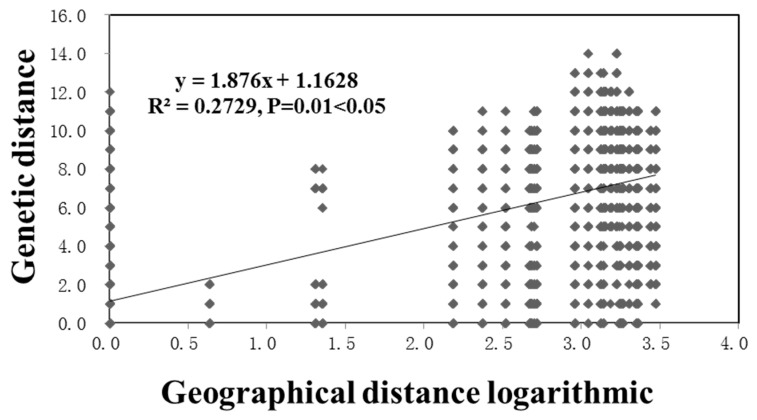
Mantel test showing relationships between genetic distance and the logarithm of geographic distance between all pairs of strains from within and between the nine local populations of *Psylliodes attenuata* from China that we analyzed here.

Results from the analyses of molecular variation (AMOVA) were consistent with those shown above based on the overall and pairwise *F_ST_* values. Specifically, geographic separation among the local populations accounted for 58% of the total genetic variance, while within-local populations accounted for 42% of the total observed genetic variance (Table 5). Furthermore, the Mantel correlation analysis between genetic distance and geographic distance showed a statistically significant positive correlation between the two distance measures (R^2^ = 0.2729, *p* = 0.01 < 0.05) (Figure 3), a result consistent with geographic separation as a significant contributor to the observed genetic structure of *P. attenuata* populations in China.

## 4. Discussion

In this study, we collected samples of *P. attenuata*, a common hemp pest, from nine geographic locations in six provinces representing the broad range of commercial hemp production in China. These hemp flea beetles were analyzed for their patterns of COI DNA sequence variation among specimens from within and between the local populations. Our results identified that 45 of the sequenced 645 bp mitochondrial COI fragments as being variable within the total population sample. These polymorphic sites separated the 281 specimens into 48 COI haplotypes. These haplotypes were present in variable frequencies, from 1/281 to 91/281. Eight of the 48 haplotypes were found in at least two of the nine local populations, and these eight haplotypes accounted for >70% of all specimens. Each of the remaining 40 haplotypes was only found in one of the nine local populations. Most haplotypes showed geographic-specific distribution patterns. The differential haplotype distributions resulted in significant population differentiation among most local populations in China. Our results provide the first survey of genetic variation on this important pest of hemp. Below we discuss our obtained results, including the implications for the management of hemp flea beetles. 

Similar to previous studies on other insects [16,28,29,30], our analyses using the mitochondrial COI gene sequences revealed abundant sequence variation among individuals and populations of the hemp flea beetle in China. In a non-model organism such as ours, the availability of a universal primer pair that allowed amplification and sequencing of a common and variable genomic region significantly facilitated our analyses. Indeed, we obtained clean COI sequences from all hemp flea beetle samples that we analyzed. The obtained COI sequences showed base compositions similar to those reported previously for mitochondrial sequences for other insects, with a heavy bias towards A/T (67.1%) [28,29]. Furthermore, because mitochondrial COI is the DNA barcode for animals [16], including insects, there is already a large number of mitochondrial COI sequences for animals deposited in GenBank and in the Barcode of Life Database (BOLD) (http://v3.boldsystems.org/index.php/databases). As a result, our data should facilitate future comparative studies with other flea beetles in the genus *Psylliodes* and other insects.

The 48 COI haplotypes showed a range of relationships with each other. Based on their sequence similarities, these haplotypes were clustered into three large groups (A, B, and C), with the B group being the intermediates of A and C groups through multiple mutational steps. Interestingly, there are several cases of haplotypes forming loops/reticulate networks. While such loops represent uncertainties in the evolutionary relationships among the observed haplotypes, the observed loop, involving haplotypes H1, H3, H6 and H16 with one mutational step between adjacent members (Figure 2), is consistent with either parallel mutations or recombination. Due to the relatively unsaturated nature of mutational targets in COI sequences (only ~7% of the sequenced fragment were polymorphic), we believe that a parallel mutation was highly unlikely to generate this loop. Instead, recombination could have happened to generate the observed haplotypes. Indeed, evidence for mitochondrial recombination has been reported in the natural populations of a variety of organisms [31,32,33].

We identified two haplotypes, H1 and H33, in very high frequencies. H1 showed a broad geographic distribution, found in central, eastern, and northern China. However, it was most frequently found in Anhui province. Its phylogenetic distribution in both the NJ tree and the network indicates that H1 was located at the root/center of many other haplotypes and likely represented a relatively ancient haplotype in the Chinese population of the hemp flea beetle. Seventeen other haplotypes differed from H1 by only one or a few mutational steps, with many of these associated haplotypes found in central and eastern China (Figure 1 and Figure 2). The result is consistent with central and eastern China as the origin of haplotype H1. In contrast, though also highly abundant, the second most common haplotype, H33, was only found in northeastern China, with most of the closely associated haplotypes also found in this region. This result is consistent with northeastern China as the origin of haplotype H33. However, for both abundant haplotypes, other closely related haplotypes were also found in broad regions, suggesting that the two common haplotypes have been accumulating mutations, and the generated new haplotypes are spreading to other geographic areas.

The presence of divergent haplotypes in different geographic regions and the observed significant genetic differentiation suggest that *P. attenuata* was likely an ancient species within China and that geographic separation has played a dominant role in the species history. The sampled regions in China also differ in their climate and other geographic conditions. Thus, it is possible that these factors have also played a role in the distribution of these haplotypes. In six of the nine local populations, as well as in the total sample, the Tajima’s D values were negative, consistent with a recent population expansion, likely after population bottlenecks. Interestingly, these COI sequence diversity results are also consistent with the recent cultivation history of hemp in China. Because of the associations with psychoactive compounds such as THC and the negative stigma about psychoactive compounds in China since the end of the opium war, hemp cultivation has been very limited and highly regulated since at least the mid-20th century, despite its long history of cultivation in China. Consequently, due to its narrow host range in favor of hemp plants, *P. attenuata* has likely experienced a population bottleneck in China since the mid-20th century. However, due to recent surging interests for both hemp fibers and its medicinal properties, hemp cultivation has been increasing rapidly. Such a rapid expansion of host hemp plants could have resulted in the rapid expansion of hemp flea beetles and the generation of many rare (singleton) haplotypes, but which are closely related to the major haplotypes such as H1 and H33. At present, the adaptive significance of the observed rare haplotypes in *P. attenuata* is not known.

Our statistical analyses of the observed COI sequence variations revealed that geographic separation played an important role in the population structure of *P. attenuata*. However, we found that certain haplotypes or closely related haplotypes were broadly distributed, consistent with long-distance dispersal or incomplete lineage sorting of this pest. Hemp flea beetles breed once a year, with their mating, feeding and living all closely associated with hemp plants. Thus, long-distance dispersal by their own flight, wind, and other natural means would be highly unlikely to account for haplotype sharing between northeastern, eastern, and central China for several of the haplotypes such as H1, H6, H7, and H24. Instead, the observed haplotype sharing among fields located thousands of kilometers apart was likely due to incomplete lineage sorting and/or recent anthropogenic activities such as human travel and trade. Indeed, anthropogenic activities have been found to impact the distributions of a variety of plant pathogens and pests [15,19,20,34,35,36]. Even for the Yunnan population of *P. attenuata*, where no haplotype was found shared with those from other geographic areas, the Yunnan haplotypes were found closely related to those in other regions separated by one or a few mutations, consistent with their recent shared histories. Indeed, more extensive sampling may reveal additional sharing of COI haplotypes among geographic populations.

Among the nine local populations, those from Shandong, Jilin and Hunan contained a high number of haplotypes, high nucleotide and haplotype diversities, and broad distributions of their haplotypes across the phylogenetic tree. Together, these results suggested that these three regions, especially Jilin, were likely the centers of *P. attenuata* diversity in China. However, additional samples of natural hemp populations and uses of DNA sequence information from other genes, including those from the nuclear genomes, are needed in order to identify the true center(s) of origins for *P. attenuata*. In China, based on our observations, the method for controlling *P. attenuata* mainly relies on chemical pesticides, including cypermethrin, abamectin and acetamiprid. In practice, farmers typically spray pesticides onto *Cannabis* seedlings to prevent *P. attenuata* from killing the young seedlings. At present, there is no measure to control this pest at later stages of *Cannabis* growth. However, as shown in our sampling, hemp flea beetles are commonly found during the late stages of *Cannabis* growth which can result in serious crop losses. Given the oligophagous and monovoltine nature of this pest, one control and prevention measure would be to use crop rotation to eliminate the food source of *P. attenuata* for one to several years, to significantly reduce or entirely eliminate the reproduction of this pest. Similarly, given the observed evidence for potential gene flow between distinct geographic populations of *P. attenuata* due to anthropogenic factors, the second measure would be to ensure that the commercial hemp seeds are free of hemp flea beetles (eggs, larvae, and adult beetles) before they are shipped from seed centers to distinct locations for commercial planting and hemp production.

## 5. Conclusions

In this study, sequences of a 645 bp fragment of the mitochondrial barcode gene COI were used to analyze the genetic diversity and structure of *P. attenuata* populations in China. Our analyses of 281 specimens identified a total of 48 haplotypes in various frequencies and distribution patterns. We found that local populations located close to each other were genetically very similar. However, regional populations from different parts of China were significantly differentiated. On the other hand, the sharing of COI haplotypes among diverse geographic regions is consistent with incomplete lineage sorting and/or recent long-distance dispersals, likely due to anthropogenic activities such as human travel and trade. The adaptive significance of the observed COI sequence variations awaits further investigation.

## Figures and Tables

**Table 1:** The details of *Psylliodes attenuata* collections.

Province	Site	Latitude/Longitude	Host Cultivar	Date Collected	Sample Collected	Population Code	Number of Samples
Anhui	Wangqiao Village	31.67/116.357	Wan *Cannabis* No. 1	July 2019	Yes	AH1	30
	Lu’an	31.806/116.519	Wan *Cannabis* No. 1	July 2019	Yes	AH2	30
	Zhangwan Village	31.70/116.358	Wan *Cannabis* No. 1	July 2019	Yes	AH3	30
Shandong	Tai’an	35.97/116.97	Zhong *Cannabis* No. 5	August 2019	Yes	SD	30
Hunan	Shijihu	28.8/112.36	Zhong *Cannabis* No. 1	August 2019	Yes	HN	42
	Yuanjiang	28.79/112.34	Zhong *Cannabis* No. 5	August 2019	No		
	Chenpo Village	28.78/112.35	Wan *Cannabis* No. 1	August 2019	No		
Yunnan	Xishuangbanna	21.99/100.41	Yunma No. 1 and 7	September 2019	No		
	Chuxiong	25.14/101.55	Yunma No. 7	September 2019	No		
	Qujing	25.85/103.75	Yunma No. 7	September 2019	Yes	YN	29
Heilongjiang	Daoli	45.59/126.44	Long *Cannabis* No. 5	September 2019	No		
	Harbin	45.59/126.44	Long *Cannabis* No. 3	September 2019	Yes	HRB	30
	Daqing	46.67/125.23	Qingma No. 1 and 2	September 2019	Yes	DQ	30
Jilin	Changchun	43.72/125.09	Fenma No. 3	September 2019	Yes	JL	30

**Table 2:** The distributions of mt-COI haplotypes of *Psylliodes attenuata* at each of nine local sites in China.

Haplotype	AH1 (30)	AH2 (30)	AH3 (30)	SD (30)	HN (42)	YN (29)	HRB (30)	DQ (30)	JL (30)	Total (281)	GenBank Accession No.
**H1**	27	25	26	8	2	0	2	1	0	91	MT447350
**H2**	1	0	0	0	0	0	0	0	0	1	MT447351
**H3**	1	0	0	0	0	0	0	0	0	1	MT447352
**H4**	1	0	0	0	0	0	0	0	0	1	MT447353
**H5**	0	1	0	0	0	0	0	0	0	1	MT447354
**H6**	0	1	0	6	7	0	0	0	0	14	MT447355
**H7**	0	1	0	5	7	0	0	1	0	14	MT447356
**H8**	0	1	0	0	0	0	0	0	0	1	MT447357
**H9**	0	1	0	0	0	0	0	0	0	1	MT447358
**H10**	0	0	2	0	0	0	0	0	0	2	MT447359
**H11**	0	0	1	0	0	0	0	0	0	1	MT447360
**H12**	0	0	1	0	0	0	0	0	0	1	MT447361
**H13**	0	0	0	1	0	0	0	0	0	1	MT447362
**H14**	0	0	0	2	0	0	0	0	1	3	MT447363
**H15**	0	0	0	1	0	0	0	0	0	1	MT447364
**H16**	0	0	0	2	0	0	0	0	0	2	MT447365
**H17**	0	0	0	2	0	0	0	0	0	2	MT447366
**H18**	0	0	0	1	0	0	0	0	0	1	MT447367
**H19**	0	0	0	1	0	0	1	0	0	2	MT447368
**H20**	0	0	0	1	0	0	0	0	0	1	MT447369
**H21**	0	0	0	0	1	0	0	0	0	1	MT447370
**H22**	0	0	0	0	2	0	0	0	0	2	MT447371
**H23**	0	0	0	0	14	0	0	0	0	14	MT447372
**H24**	0	0	0	0	5	0	1	4	0	10	MT447373
**H25**	0	0	0	0	3	0	0	0	0	3	MT447374
**H26**	0	0	0	0	1	0	0	0	0	1	MT447375
**H27**	0	0	0	0	0	8	0	0	0	8	MT447376
**H28**	0	0	0	0	0	6	0	0	0	6	MT447377
**H29**	0	0	0	0	0	7	0	0	0	7	MT447378
**H30**	0	0	0	0	0	5	0	0	0	5	MT447379
**H31**	0	0	0	0	0	2	0	0	0	2	MT447380
**H32**	0	0	0	0	0	1	0	0	0	1	MT447381
**H33**	0	0	0	0	0	0	22	19	20	61	MT447382
**H34**	0	0	0	0	0	0	1	0	0	1	MT447383
**H35**	0	0	0	0	0	0	2	0	0	2	MT447384
**H36**	0	0	0	0	0	0	1	0	0	1	MT447385
**H37**	0	0	0	0	0	0	0	0	1	1	MT447386
**H38**	0	0	0	0	0	0	0	0	1	1	MT447387
**H39**	0	0	0	0	0	0	0	1	2	3	MT447388
**H40**	0	0	0	0	0	0	0	0	1	1	MT447389
**H41**	0	0	0	0	0	0	0	0	1	1	MT447390
**H42**	0	0	0	0	0	0	0	0	1	1	MT447391
**H43**	0	0	0	0	0	0	0	0	1	1	MT447392
**H44**	0	0	0	0	0	0	0	0	1	1	MT447393
**H45**	0	0	0	0	0	0	0	1	0	1	MT447394
**H46**	0	0	0	0	0	0	0	1	0	1	MT447395
**H47**	0	0	0	0	0	0	0	1	0	1	MT447396
**H48**	0	0	0	0	0	0	0	1	0	1	MT447397

**Table 3:** Genetic diversity of mt-COI haplotypes within the nine local populations of the hemp flea beetle *Psylliodes attenuata* in China.

Population Code	Number of Haplotypes (No. of Private Haplotypes)	Haplotype Diversity (Hd)	Nucleotide Diversity (π)	Tajima’s D
**AH1**	4 (3)	0.193	0.00031	−1.73178 ^NS^
**AH2**	6 (3)	0.310	0.00114	−2.36834 **
**AH3**	4 (3)	0.251	0.00041	−1.53889 ^NS^
**SD**	11 (6)	0.871	0.00688	0.11613 ^NS^
**HN**	9 (5)	0.828	0.00982	1.41773 ^NS^
**YN**	6 (6)	0.815	0.00600	1.23395 ^NS^
**HRB**	7 (3)	0.464	0.00342	−1.08139 ^NS^
**DQ**	9 (4)	0.593	0.00274	−2.01006 *
**JL**	10 (7)	0.561	0.00260	−1.98693 *
**Total**	48 (40)	0.839	0.00871	−0.74006 ^NS^

*, *p* < 0.05; **, *p* < 0.01; ^NS^, statistically not significant.

**Table 4:** Pairwise *F_ST_* values between local populations of *Psylliodes attenuata* in China.

	AH2	AH3	SD	HN	YN	HRB	JL	DQ
**AH1**	−0.01205	0.00985	0.27120 **	0.50895 **	0.78838 ***	0.83614 ***	0.88233 ***	0.87890 **
**AH2**		0.00460	0.22032 **	0.47284 **	0.76146 ***	0.80146 ***	0.84955 ***	0.84681 ***
**AH3**			0.28079 **	0.51317 **	0.78834 ***	0.83467 ***	0.88037 ***	0.87709 ***
**SD**				0.16657 *	0.52979 **	0.52815 **	0.58310 **	0.58559 **
**HN**					0.32704 **	0.33311 **	0.37098 **	0.36893 **
**YN**						0.28706 **	0.31985 **	0.28882 **
**HRB**							0.02170	0.00353
**JL**								0.02226
**DQ**								

*, *p* < 0.05; **, *p* < 0.01; ***, *p* < 0.001.

**Table 5:** Analysis of molecular variance within and among local populations *Psylliodes attenuata* from China.

Source of Variation	df	Sum of Squares	Variance Components	Variation Proportion
Among populations	8	475.802	1.866	58%
Within populations	272	363.586	1.337	42%
Total	280	839.388	3.202	100%
